# Characterisation at Cryogenic Temperatures of an Attenuator for an Application of Astrophysical Instrumentation with MKIDs

**DOI:** 10.3390/s24248129

**Published:** 2024-12-19

**Authors:** Diego Portero-Rodríguez, Hugo García-Vázquez, José Luis Martínez-Rodríguez, Sergio Elías Hernández Alonso, Enrique Joven Álvarez, Roger John Hoyland, José Javier Díaz García, Luis Fernando Rodríguez Ramos

**Affiliations:** 1 Laboratorio de Circuitos Integrados (LABIC), Departamento de Electrónica, Área de Instrumentación, Instituto de Astrofísica de Canarias (IAC), 38205 La Laguna, Tenerife, Spain; 2 Departamento de Astrofísica, Universidad de La Laguna (ULL), 38206 La Laguna, Tenerife, Spain; 3 Departamento de Ingeniería Industrial, Universidad de La Laguna (ULL), 38206 La Laguna, Tenerife, Spain

**Keywords:** cryogenic, vacuum, astrophysical instrumentation, integrated circuit (IC), attenuator, microwaves, non-cryogenic certified components, MKIDs

## Abstract

The use of non-cryogenic certified commercial electronics for cryogenic applications may be attractive due to their cost and availability, but it also carries risks related to reliability, performance and thermal compatibility. The decision to use commercial components that are not certified for cryogenics instead of components specifically designed for such applications must be carefully weighed based on specific project needs and risk tolerances. This work presents the characterisation of an attenuator circuit at cryogenic temperatures used in a microwave kinetic inductance detector (MKID) readout system. In order to characterise the operation of the attenuator at cryogenic temperatures and because the circuit works at frequencies up to 40 GHz, a specific microwave PCB has been designed. The cooling system used consists of a cryostat, all the connectors, cables, a vacuum pump, a compressor, pressure and temperature sensors, a temperature control system and a cold head operating in a closed helium gas cycle according to the Gifford–McMahon principle. The circuit was tested and characterised at temperatures ranging from 296.5 K to 83.6 K.

## 1. Introduction

Microwave kinetic inductance detectors (MKIDs) represent an emerging type of detector that has been proven to be useful in a range of applications [1,2]. In the field of astronomy, there are many applications [3,4,5] and instruments, such as the NIKA2 large-field-of-view millimetre continuum camera in the IRAM telescope [6], the MISTRAL instrument in the Sardinia Radio Telescope (SRT) [7], the TolTEC camera in the Large Millimetre Telescope (LMT) Alfonso Serrano [8] or the updated design of the SPT-3G+ camera for the South Pole Telescope (SPT) [9].

MKIDs consist of a chain of resonators made of superconducting materials operated at sub-Kelvin (K) temperatures that are used for high-sensitivity astronomical applications. The working principle of these detectors is based on the principle that the surface impedance of a superconductor is modified by incident photons. An incident photon with an energy level of hν>2Δ, where Δ is the superconducting gap energy in a superconducting film, causes the breaking of Cooper pairs and creates a number of excitations, known as quasi-particles [1,10,11].

A general and open-source configuration for performing the readout from these devices has already been proposed [12,13,14,15,16]. In these systems, a control and signal processing unit generates a comb of probe frequencies for each resonator, which are then digitised and sent to a filtering and mixing stage. This signal is then sent to the component on which this work focuses: an attenuator. The main reason for placing an attenuator before the MKID is that a high signal level (for a high signal-to-noise ratio) will increase the latter’s temperature and so must be attenuated before reaching the MKID.

**Figure 1:**
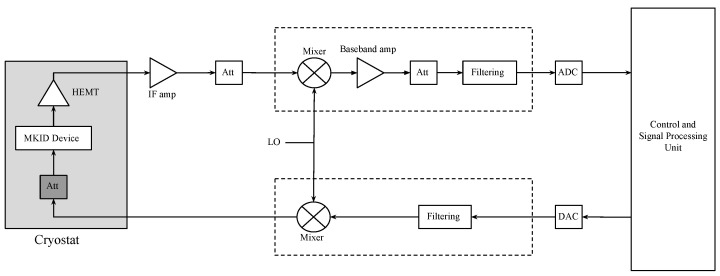
Proposed MKID readout configuration.

A schematic of the proposed readout configuration of this work is depicted in Figure 1. The MKID is placed on the coldest stage of the cryostat, and the attenuator is placed just before the MKID on a different stage at a temperature of around 80 K. This is because the signal attenuation is such that the resistive noise emitted from the attenuator is now significant compared to the input signal. At room temperature (RT = 300 K), this thermally generated noise is sufficiently high as to dominate the phase readout of the detector. Cooling the attenuator to <4 K virtually removes this added signal, and the phase readout is then dominated by other parameters. Even around 80 K, the noise from the attenuator is improved by a factor of 4 with respect to 300 K.

In the field of astrophysical instrumentation, there are a large number of electronic components that are subjected to cryogenic temperatures [17,18,19,20,21,22,23,24,25]. However, most of the electronic components available on the market are not usually prepared or characterised to work at cryogenic temperatures. In most cases, the minimum working temperature given by the manufacturer does not drop below 0, −40 or −55 °C for commercial, industrial and military electronics, respectively. These minimum temperatures are well above the ranges needed in cryogenics, which are below 120 K [23,26,27].

In certain instances, the minimum working temperature provided by the manufacturer does not imply that the component cannot work at lower temperatures, but rather that it is only characterised for commercial, industrial or military ranges. Using this type of electronics for cryogenic applications can result in huge savings in component costs, as the price of electronics specially designed for cryogenics is extremely high.

**Figure 2:**
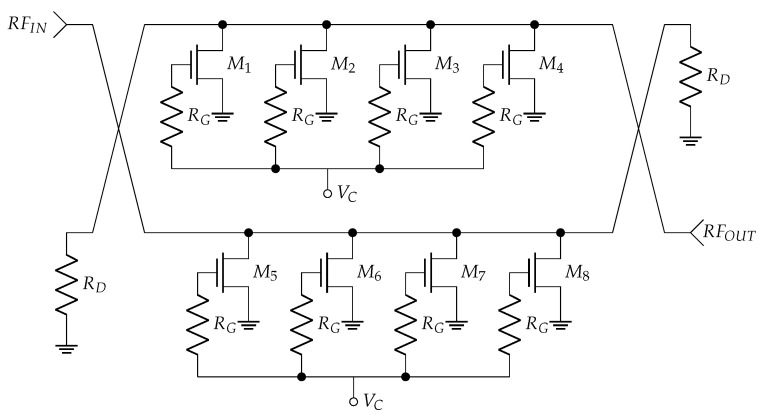
Schematic of the attenuator.

The AV850M1-00 [28] monolithic microwave integrated circuit (MMIC) is a commercial voltage variable attenuator that exhibits a maximum working frequency of 40 GHz and an attenuation range of 40 dB. The chip was designed using commercial 0.25 μm technology, and it was characterised for temperatures between −55 °C and 90 °C. Figure 2 shows the schematic of the attenuator. The attenuation range can be controlled by the control voltage ($V_{C}$) and incorporates Lange couplers [29,30,31] at its input and output. The $V_{C}$ was set to 0 V to ensure maximum attenuation. This work presents the characterisation of the circuit at cryogenic temperatures to be able to use it in an astrophysical instrumentation application with MKIDs.

The paper is structured as follows: First, in the introduction, the MKIDs and the requirement of working under cryogenic conditions are described, and the proposed attenuator is presented. Subsequently, in Section 2, the chip testing platform used to characterise the circuit is explained in detail. In the third section, measurements of the attenuator are conducted at the temperatures of interest, and the experimental results are presented. Finally, Section 4 concludes with the key findings of this work.

## 2. Chip Testing Platform

### 2.1. PCB Design

The PCB was designed with the open-source electronics design automation suite KICAD using a 250 μm thick Rogers RT/duroid^®^ 5880 substrate [32,33], commonly used for high-frequency design applications [34,35,36,37,38]. Table 1 shows the value of the different parameters used for calculating the length, width and losses of the microstrip and the results obtained using the equations described in [29], where $\epsilon_{r}$ represents the dielectric constant, tan(δ) is the dissipation factor, $\mu_{0}$ is the vacuum magnetic permeability, σ is the copper electrical conductivity, and Φ is the phase difference across the microstrip.

**Figure 3:**
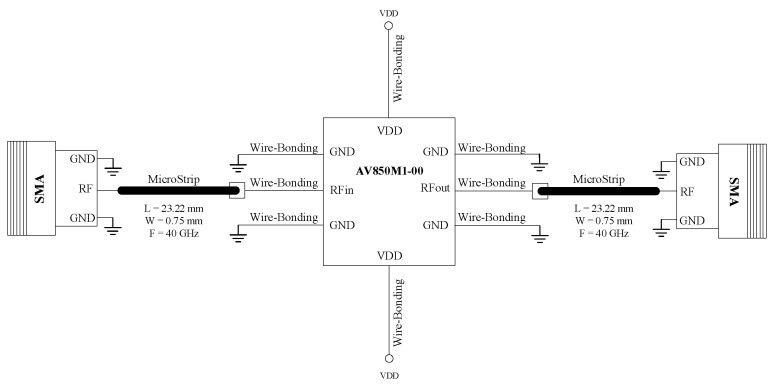
Schematic of the implemented PCB.

**Figure 4:**
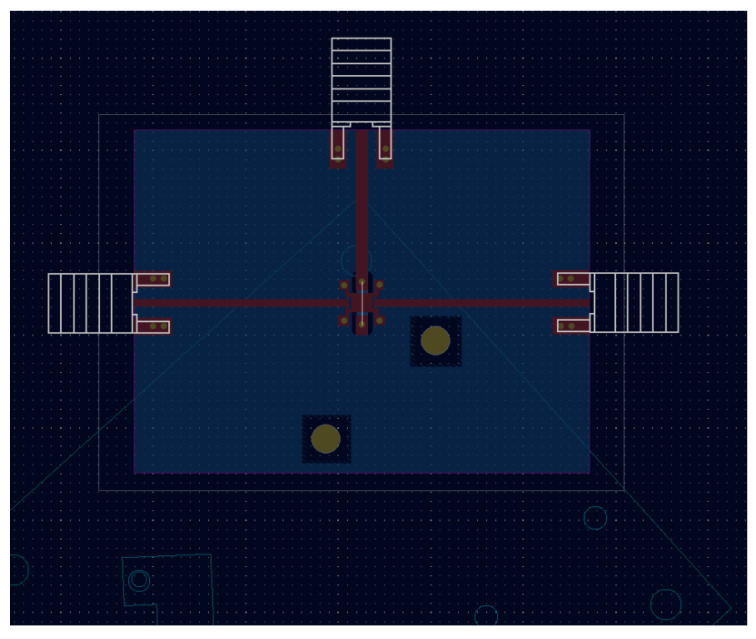
Layout of the implemented PCB.

Figure 3 and Figure 4 show the schematic and the layout of the PCB. It depicts how all the wire bonds and microstrips are placed to interconnect the PCB, the integrated circuit (IC), SMA connectors and the RF_IN_, RF_OUT_, V_DD_ and GND signals.

**Figure 5:**
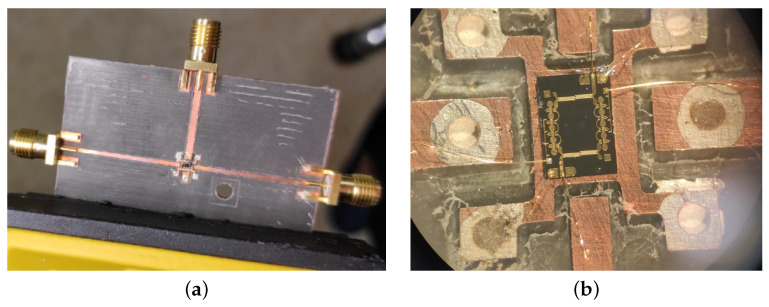
(**a**) PCB and (**b**) wire bonds between the IC and the PCB.

Figure 5a illustrates the manufactured PCB, including the chip, connectors and wire bonds. In Figure 5b, a magnified view of the wire bonds between the attenuator and the PCB is shown.

### 2.2. Cryogenic System

The cooling system consists of a cryostat with a cold head operating in a closed helium gas cycle according to the Gifford–McMahon principle [39,40], a compressor, connectors, cables, cable glands, a vacuum pump, pressure and temperature sensors and a temperature control system. The circuit behaviour was analysed at temperatures ranging from 296.5 K to 83.6 K.

**Figure 6:**
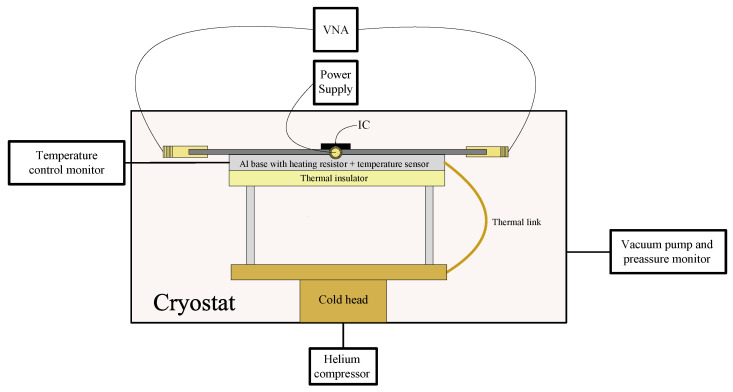
Setup used to measure the IC.

**Figure 7:**
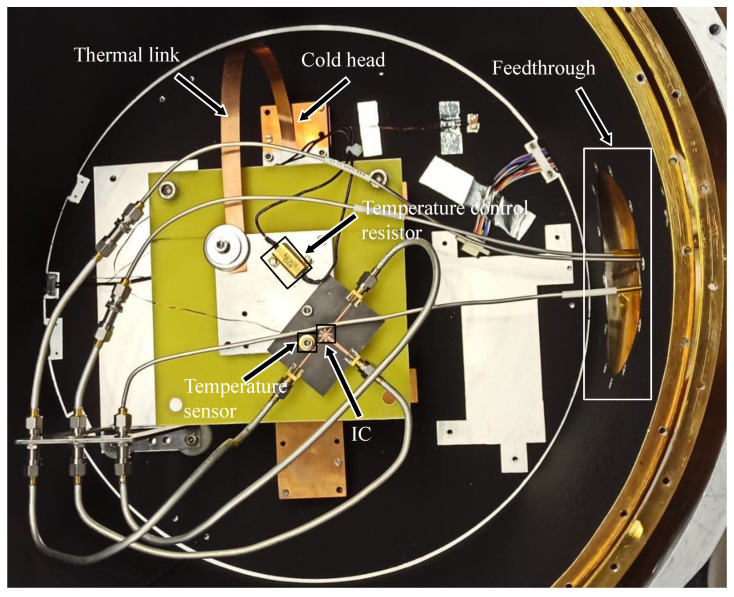
Photograph of the cryostat setup for the test.

Figure 6 shows the setup used to measure the IC. To control the temperature, a heating resistor was placed on the same surface as the PCB. As direct heating of the cold head could damage it, a thermal link was made to the surface where all the components were placed. A temperature sensor was placed as close as possible to the IC to ensure that the temperature reading was approximately the same as the IC temperature. The S-parameters of the circuit were measured using a two-port Vectorial Network Analyser (VNA) with a frequency range from 9 kHz to 20 GHz. A temperature control and monitor system was employed to monitor the temperature sensor and to control the heating resistor. Figure 7 shows a photograph of the cryostat before closing it for the tests.

**Figure 8:**
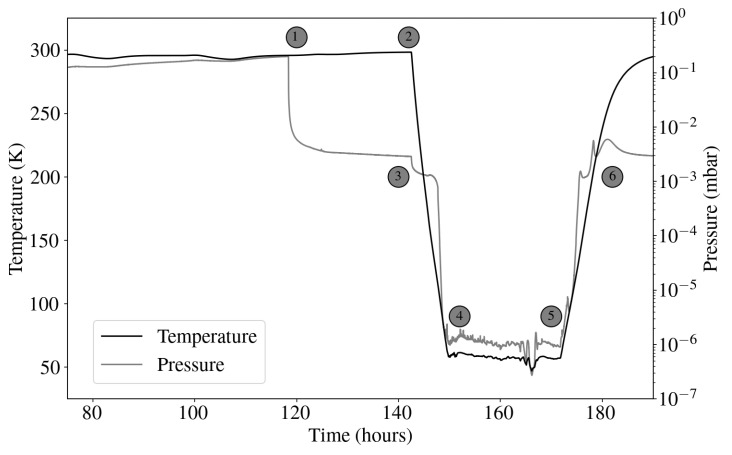
Evolution of pressure and temperature inside the cryostat without PCB or cables.

Figure 8 shows the evolution of pressure inside the cryostat and temperature at the coolest stage, without the elements shown in Figure 7. At point 1, the cryostat remains at a constant pressure level until the vacuum pump is switched on. When this happens, the pressure starts to fall until the pressure in the cryostat reaches the pressure level in the vacuum pump, at about $10^{-3}$ mbar. The temperature inside the cryostat remains at room temperature until the compressor is switched on (point 2). At this point, the cold head starts to work, and the temperature inside the cryostat starts to fall. After three hours, the temperature of the first stage is about 150 K, and the pressure inside the cryostat is lower than in the vacuum pump. At point 3, the valve between the vacuum pump and the cryostat is closed, and the vacuum pump is turned off, causing both the temperature and pressure to drop drastically. At point 4, a stable pressure level ($10^{-6}$ mbar) and cryogenic temperature (60 K) are reached. Once the measurement is completed, the compressor can be switched off (point 5), causing the pressure and the temperature to rise (point 6).

## 3. Results and Discussion

**Figure 9:**
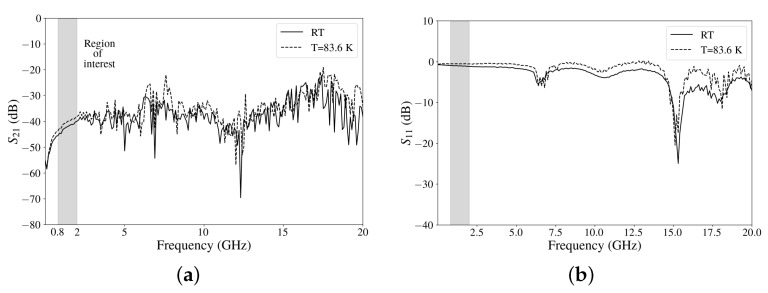
Experimental results at room temperature and the lowest temperature (83.6 K): (**a**) $S_{21}$ and (**b**) $S_{11}$.

When the measurements were carried out with the attenuator inside, the minimum temperature obtained was 83.6 K. This is due to the thermal gradient produced for the connections of the RF cables (model AM10-SR141TP-AM10) between the SMA feed-through at the external wall of the cryostat (at room temperature) and the PCB. Figure 9 shows the results of the attenuator at room temperature (RT) and the minimum temperature achieved using the chip testing platform. The $S_{21}$ shows an attenuation higher than 20 dB for the whole frequency range. An $S_{11}$ close to 0 dB is obtained for most of the frequency range. In the interest frequency band for our application (0.8–2 GHz), an attenuation between −40.5 and −45.5 dB was obtained for room temperature and one of between −38 and −43.5 dB for a temperature of 83.6 K.

**Figure 10:**
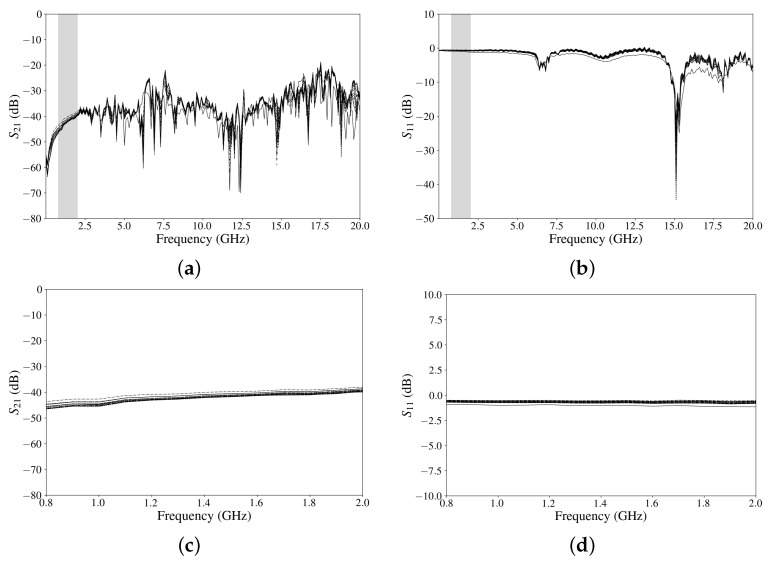
Experimental results from 296.5 K to 83.6 K: (**a**) $S_{21}$, (**b**) $S_{11}$, (**c**) $S_{21}$ (region of interest) and (**d**) $S_{11}$ (region of interest).

**Figure 11:**
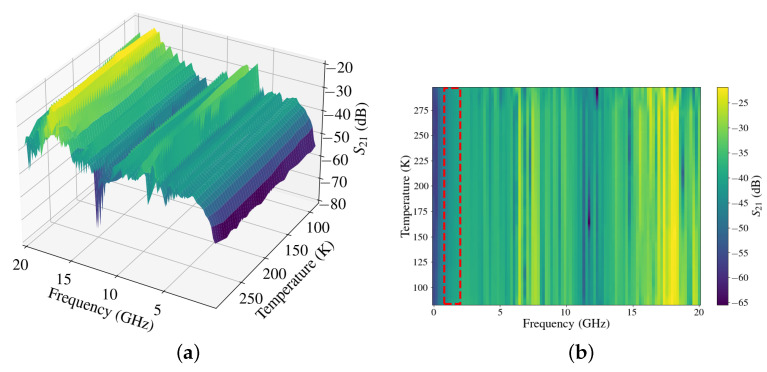
Experimental results from 296.5 K to 83.6 K: (**a**) 3D plot and (**b**) colour map.

The circuit was tested and characterised for different temperatures ranging from 296.5 K to 83.6 K (21 temperature steps), as shown in Figure 10. As can be seen, the values of $S_{11}$ and $S_{21}$ do not vary significantly and follow a general trend. Figure 11 shows a 3D plot and a colour map, which illustrate that the attenuation does not vary significantly with the temperature in the range of interest. Figure 11a shows a 3D plot where the generation of valleys in the attenuation (from −20 to −70 dB) at high frequencies (above 5 GHz) are appreciated. Below that frequency, the attenuation remains between −30 and −50 dB. Figure 11b depicts a grey colour map of the 3D plot, and it shows a good response against changes in temperature for frequency ranges between 800 MHz and 2 GHz.

**Figure 12:**
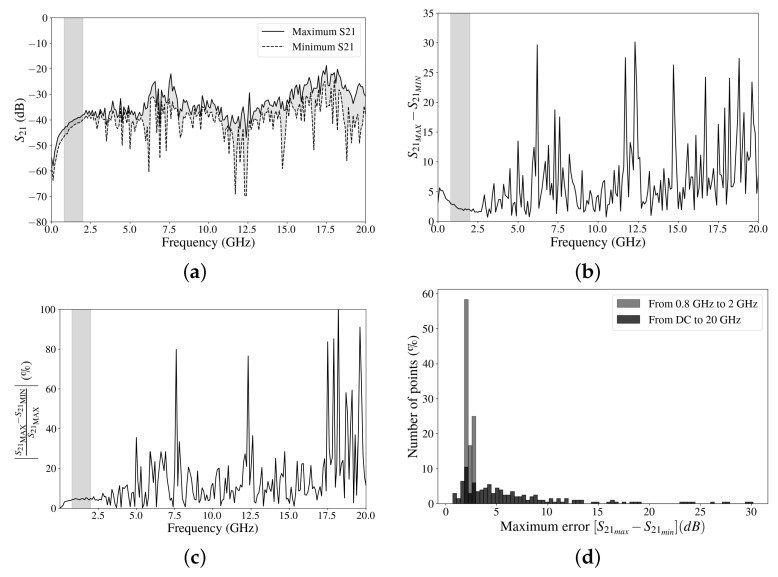
Experimental results from 296.5 K to 83.6 K: (**a**) $S_{21_{MAX}}$ and $S_{21_{MIN}}$, (**b**) $S_{21_{MAX}}-S_{21_{MIN}}$ in absolute values, (**c**) $S_{21_{MAX}}-S_{21_{MIN}}$ in percentages with $S_{21_{MAX}}$ as a reference and (**d**) histogram.

In order to see the maximum differences that can be obtained for the range of temperatures across the complete bandwidth, two curves with the maximum and minimum values are plotted in Figure 12a. It can be clearly appreciated how there are frequency ranges in which higher differences in the attenuation value occur. Figure 12b,c represent these differences in the attenuation across the frequencies in absolute values and percentages, respectively. Although, in many cases, the variations are less than 5 dB, there are some frequency ranges in which the difference between the maximum and minimum values can reach up to 30 dB. A histogram representing the dispersion of the $S_{21_{MAX}}-S_{21_{MIN}}$ difference is depicted in Figure 12d. The standard deviation (σ) is a commonly employed parameter for quantifying data dispersion. For the entire frequency range, a standard deviation of 5.84 dB and an arithmetic mean ($\bar{x}$) of 6.55 dB were obtained. In contrast, within the range of interest, these values drop to a σ of 0.39 dB and a $\bar{x}$ of 2.27 dB.

Therefore, with the results obtained, it is necessary to be cautious with regard to the working frequency, since although, in general, it has flat attenuation and very good performance, there are some frequency ranges in which the attenuation value can vary considerably. In terms of our application, the frequency range of interest (0.8–2 GHz) has differences in the attenuation smaller than 2 dB produced for the different temperatures. Therefore, for the whole range of interest, the attenuation differences are lower than 5.5 dB at the lowest temperature, which are adequate and do not negatively affect the operation of the system.

## 4. Conclusions

As discussed previously, many instruments used in astronomy need to be cooled to cryogenic temperatures. In particular, this work analysed the features in cryogenics of a non-certified-for-cryogenics commercial integrated attenuator designed with a 0.25 μm technology for an astronomy application with MKIDs. The results show how, at temperatures ranging from 296.5 K to 83.6 K, this integrated circuit continued working adequately. However, although the results are generally satisfactory, there are certain frequency ranges in which the response is poor. Therefore, to obtain an adequate result, it is important to correctly choose the frequency range. The use of non-certified components for cryogenics can result in a huge saving in costs, but their use must be carefully weighed based on the specific project requirements and necessities.

## Figures and Tables

**Table 1:** Microstrip parameters.

Substrate		Requirements		Results	
**Parameter**	**Value**	**Parameter**	**Value**	**Parameter**	**Value**
Laminate	Rogers RT/duroid^®^ 5880	Frequency (GHz)	40	Dielectric losses $\alpha_{d}$ (dB)	0.086
$\epsilon_{r}$	2.2	Z, Impedance (Ω)	50	Conductor losses $\alpha_{c}$ (dB)	0.00022
tan(δ)	0.0009	Φ (°)	90	Length, L (mm)	23.22
d (mm)	0.25			Width, W (mm)	0.75
$\mu_{0}$ (Tm/A)	$4\pi \times 10^{-7}$				
σ (S/m)	5.8$\times 10^{7}$				

## Data Availability

The data are contained within the article. Further inquiries can be directed to the corresponding author.
